# Adipokines: New Potential Therapeutic Target for Obesity and Metabolic, Rheumatic, and Cardiovascular Diseases

**DOI:** 10.3389/fphys.2020.578966

**Published:** 2020-10-30

**Authors:** Lucia Recinella, Giustino Orlando, Claudio Ferrante, Annalisa Chiavaroli, Luigi Brunetti, Sheila Leone

**Affiliations:** Department of Pharmacy, Gabriele d’Annunzio University, Chieti, Italy

**Keywords:** adipokines, obesity, type 2 diabetes mellitus, cardiovascular disorders, therapeutic targets, rheumatoid arthritis

## Abstract

Besides its role as an energy storage organ, adipose tissue can be viewed as a dynamic and complex endocrine organ, which produces and secretes several adipokines, including hormones, cytokines, extracellular matrix (ECM) proteins, and growth and vasoactive factors. A wide body of evidence showed that adipokines play a critical role in various biological and physiological functions, among which feeding modulation, inflammatory and immune function, glucose and lipid metabolism, and blood pressure control. The aim of this review is to summarize the effects of several adipokines, including leptin, diponectin, resistin, chemerin, lipocalin-2 (LCN2), vaspin, omentin, follistatin-like 1 (FSTL1), secreted protein acidic and rich in cysteine (SPARC), secreted frizzled-related protein 5 (SFRP5), C1q/TNF-related proteins (CTRPs), family with sequence similarity to 19 member A5 (FAM19A5), wingless-type inducible signaling pathway protein-1 (WISP1), progranulin (PGRN), nesfatin-1 (nesfatin), visfatin/PBEF/NAMPT, apelin, retinol binding protein 4 (RPB4), and plasminogen activator inhibitor-1 (PAI-1) in the regulation of insulin resistance and vascular function, as well as many aspects of inflammation and immunity and their potential role in managing obesity-associated diseases, including metabolic, osteoarticular, and cardiovascular diseases.

## Introduction

Adipose tissue is currently considered as an endocrine organ, a complex structure involved not only in fat storage but also in releasing several bioactive polypeptides, collectively named “adipokines” (Kershaw and Flier, 2004; Galic et al., 2010). These adipokines are able to modulate body weight, appetite, glucose homeostasis, inflammation, and blood pressure (Hida et al., 2005; Trujillo and Scherer, 2006; Rabe et al., 2008; Mancuso, 2016; Wang and Scherer, 2016; Weschenfelder et al., 2020). On the other hand, hormones produced in different organs, such as the gut and the cardiovascular system, are integrated in a complex network centered in adipose tissue (Kershaw and Flier, 2004; Galic et al., 2010).

Adipokines are released by either adipocytes (the most abundant being leptin and adiponectin) or preadipocytes, adipose tissue-infiltrated immune cells, or other cell types within adipose tissue. Moreover, adipocytes are also able to produce proinflammatory cytokines, particularly in the chronic subinflammatory state of obesity, and antinflammatory cytokines (Villarroya et al., 2018). In addition, the various immune cell types within adipose tissue contribute to this release. In healthy individuals, type 2 innate lymphoid cells and other Th2 immune cells promote adipose glucose homeostasis by enhancing insulin sensitivity in adipocytes and browning/brown adipose tissue (BAT) activation. On the other hand, pathogenic inflammation of white adipose tissue (WAT) leads to the activation of pro-inflammatory pathways in adipocytes and resident immune cells following obesity, enhancing the production of pro-inflammatory, and inhibiting release of antinflammatory cytokines and adipokines (Villarroya et al., 2018). Recently, it has also been demonstrated that local pro-inflammatory signaling in BAT could repress the thermogenic function thereby impairing diet-induced thermogenesis (Villarroya et al., 2018). All together, these adipose tissue derived factors could be critically involved in the development of insulin resistance, type 2 diabetes mellitus (T2DM), and cardiovascular disease associated with obesity, as reviewed by various authors (Antuna-Puente et al., 2008; Cao, 2014; Nakamura et al., 2014).

More recently, other adipokines such as chemerin, lipocalin-2 (LCN2), vaspin, and omentin-1 have been described as signal molecules involved in neuroendocrine-immune interactions (Carrión et al., 2019; Xie and Chen, 2019). Besides adipose tissue, these adipokines can be produced by synoviocytes, osteoclasts, osteoblasts, chondrocytes, and inflammatory cells which have migrated in the joint microenvironment, and show potent immunomodulatory activities in rheumatic disease, such as in osteoarthritis (OA) and rheumatoid arthritis (RA) (Carrión et al., 2019; Xie and Chen, 2019). In addition, novel adipokines such as follistatin-like 1 (FSTL1), secreted protein acidic and rich in cysteine (SPARC), secreted frizzled-related protein 5 (SFRP5), C1q/TNF-related proteins (CTRPs), as well as family with sequence similarity to 19 member A5 (FAM19A5), wingless-type inducible signaling pathway protein-1 (WISP1), progranulin (PGRN), nesfatin-1 (nesfatin), visfatin/PBEF/NAMPT, apelin, retinol binding protein 4 (RPB4), and plasminogen activator inhibitor-1 (PAI-1) have been recently identified in adipose tissue. However, the mechanisms underlying the molecular and physiological actions of these adipokines are not entirely clear, and further investigations are needed to develop novel therapeutic agents directed to these targets. In this review, we aimed to summarize the effects of adipokines in the regulation of insulin resistance and vascular function, as well as inflammation and immunity and their potential role in managing obesity-associated diseases, including metabolic, rheumatic, and cardiovascular disorders (Table 1).

**TABLE 1:** Summary of effects of known adipokines on obesity and metabolic, rheumatic, and cardiovascular diseases.

Adipokine	Obesity and metabolic dysfunction	Rheumatic diseases	Cardiovascular disease
**Leptin**	Hyperleptinemia is positively correlated with obesity and metabolic dysfunction, decreases food intake, increases energy expenditure and insulin resistance (Rosenbaum and Leibel, 1999; Ahima et al., 2000; Schwartz et al., 2000; Hafizuallah, 2006; Al-Amodi et al., 2018)	Hyperleptinemia is associated with erosions in patients with RA (Lee et al., 2007; Yoshino et al., 2011; Olama et al., 2012; Cao et al., 2016; Lee and Bae, 2016)	Hyperleptinemia is associated with protrombotic effects, endothelial dysfunction, cardiovascular remodeling; leptin regulates cardiac contractile function, metabolism, cell size, and production of extracellular matrix components in cardiomyocytes (Beltowski, 2006; Momin et al., 2006; Adiarto et al., 2007; Katsiki et al., 2018)
**Adiponectin**	Hypoadiponectinemia is associated with obesity, insulin resistance, T2DM, and metabolic syndrome (Arita et al., 1999; Hotta et al., 2000; Weyer et al., 2001; Mohan et al., 2005; Gilardini et al., 2006; Yamauchi et al., 2007; Wang et al., 2008)	Hyperadiponectinemia was found in chronic inflammatory RA (Neumann et al., 2011; Fatel et al., 2018)	Hypoadiponectinemia is associated with hypertension, endothelial dysfunction, elevated myocardial hypertrophy (Diah et al., 2019)
**Resistin**	Hyper-resistinemia is associated with obesity and insulin resistance (Rajala et al., 2004; Rangwala et al., 2004; Schäffler et al., 2004; Vendrell et al., 2004; Satoh et al., 2014)	Higher resistin levels were found in serum and synovial fluid of RA patients. It accumulates in the inflamed joints of RA patients inducing the development of arthritis accompanied by leukocytic infiltration and hyperplasia of the synovium (Kassem et al., 2010)	Hyper-resistinemia is associated with cardiovascular risk, endothelial dysfunction, and hypertension (Takata et al., 2008; Zhang L. et al., 2010; Thomopoulos et al., 2011)
**Chemerin**	Hyperchemerinemia is positively associated with markers of inflammation and components of the metabolic syndrome. Possible link between chemerin, obesity, and T2DM development. Chemerin stimulates insulin sensitivity in adipose tissue (Bozaoglu et al., 2007; Ernst et al., 2010)	Chemerin is present in synovial fluids of RA and OA, stimulates the leukocyte migration into the joint and the pro-inflammatory markers in synovial fibroblasts from RA patients (Buechler, 2014; Fatima et al., 2014; Mariani and Roncucci, 2015)	Hyperchemerinemia is associated with cardiovascular risks in patients with metabolic syndrome (Wu et al., 2020)
**LCN2**	LCN2 is associated with low level systemic inflammation in metabolic syndrome and obese patients (Jang et al., 2012; Abella et al., 2015)	Higher LCN2 concentrations in SF of RA patients (Gupta et al., 2007; Katano et al., 2009; Staikos et al., 2013)	Higher LCN2 levels are significantly associated with heart failure, coronary heart disease, and stroke (Hemdahl et al., 2006; Folkesson et al., 2007; Sivalingam et al., 2017)
**Vaspin**	Higher serum vaspin levels in subjects with obesity and T2DM (Feng et al., 2014)	Low serum vaspin concentration in OA patients (Bao et al., 2014) Higher serum vaspin levels in psoriatic arthritis and RA patients (Ozgen et al., 2010; Colak et al., 2019)	Low vaspin levels are associated with coronary artery disease severity (Kadoglou et al., 2011)
**Omentin-1**	Low omentin-1 levels are associated with obesity. Omentin-1 enhances the effect of insulin on glucose metabolism (de Souza Batista et al., 2007)	Hypo-omentinemia is associated with chronic inflammatory RA (Senolt et al., 2010) Low omentin-1 concentrations in SF are detectable in patients with RA (Li Z. G. et al., 2012)	Omentin-1 concentrations are inversely correlated with cardiovascular disease thanks its vasodilating effect (Tsuji et al., 2001; Yamawaki et al., 2010, 2011; Duan et al., 2011)
**FSTL1**	Overweight and mild obesity might be associated with increased FSTL1 levels (Horak et al., 2018)	Controversial data (FSTL1 promotes and/or inhibits inflammation in CAIA model) (Mattiotti et al., 2018)	Higher FSTL1 circulating concentrations were found in patients with heart failure and acute coronary syndrome (Oshima et al., 2008)
**SPARC**	Overexpression of SPARC modulates the expression levels of various pro-inflammatory cytokines, critically involved in insulin resistance, glucose, and lipid metabolism during adipogenesis (Termine et al., 1981; Shen et al., 2014)	Higher SPARC levels in synovial fibroblasts from patients with RA or OA (Bradshaw et al., 2003; Delany et al., 2003; Robey and Boskey, 2003)	SPARC-induced positive effect in the acute phase of myocardial infarction (Myasoedova et al., 2018)
**SFRP5**	Plasma concentrations of SFRP5 are unfavorably correlated with obesity (Schulte et al., 2012)	SFRP5 suppresses the inflammatory response in fibroblast-like synoviocytes of RA patients, inhibiting WNT signaling (Kwon et al., 2014)	Serum SFRP5 levels are inversely related to coronary artery disease, development of atherosclerosis, and vascular function (Safoura and Gholam, 2018)
**CTRPs**	Higher CTRP6 levels in obesity (Lei et al., 2017)	CTRP3 promotes proliferation of chondrocytes (Maeda et al., 2006)	CTRP3 is involved in progressive remodeling after myocardial infarction, exerts vasoprotective effects (Schäffler and Buechler, 2012)
**FAM19A5**	FAM19A5 concentrations were significantly elevated in patients with T2DM (Tourniaire et al., 2013)	Studies on rheumatic disease are limited	FAM19A5 prevented post-injury neointima formation in rat carotid arteries and mouse femoral arteries *in vivo* (Wang et al., 2018)
**WISP1**	Higher WISP1 circulating levels in obese and insulin resistance patients (Sahin Ersoy et al., 2016)	High WISP1 levels are harmful to cartilage integrity in osteoarthritic patients (van den Bosch et al., 2019)	WISP1 regulates cardiac endothelial signaling and contributes to beneficial effects in MI (Price et al., 2004; Reddy et al., 2011; Liu et al., 2013)
**PGRN**	Hyperprogranulinemia is associated with obesity-associated insulin resistance (Youn et al., 2009; Qu et al., 2013; Shafaei et al., 2016)	Hyperprogranulinemia is associated with RA (Cerezo et al., 2015; Abella et al., 2016)	PGRN improves tissue repair by active recruitment of leukocytes and increasing capillary perfusion after acute injury (Jian et al., 2012)
**Nesfatin-1**	Low serum nesfatin-1 levels were found in obesity and metabolic syndrome (Gonzalez et al., 2011)	Positive association between blood serum nesfastin-1 levels and CRP in RA patients (Kvlividze et al., 2019)	Decreased plasma nesfatin-1 levels in patients with acute myocardial infarction (Ramesh et al., 2017)
**Visfatin/PBEF/NAMPT**	Hypervisfatinemia is associated with obesity, T2DM, and metabolic syndrome (Chang et al., 2011)	Higher serum visfatin levels in RA patients (Meier et al., 2012; Neumann et al., 2016)	Hypervisfatinemia is associated with cardiovascular disease (Xiao et al., 2009; Anfossi et al., 2010)
**Apelin**	Higher apelin levels in obese patients (Heinonen et al., 2005; Schinzari et al., 2017; Bertrand et al., 2018)	Lower apelin levels in early stage RA patients (Di Franco et al., 2012)	Hypoapelinemia is associated with severe chronic heart failure (Chong et al., 2006)
**RBP4**	RBP4 levels were positively associated with BMI (Aeberli et al., 2007; Haider et al., 2007; Jia et al., 2007; Klöting et al., 2007)	Elevated RBP4 serum levels are associated with increased risk of insulin resistance in patients with early and untreated RA (Wei et al., 2019)	Higher serum RBP4 levels in patients with cardiovascular diseases, atherosclerosis, and hypertension (Rychter et al., 2020)
**PAI-1**	PAI-1 levels increase in subjects with obesity, metabolic syndrome, and T2DM (Vague et al., 1986; Juhan-Vague et al., 2003; Bilgili et al., 2008)	Elevated PAI-1 levels in patients with SLE but no in RA (Bae and Lee, 2020)	Elevated plasma PAI-1 concentrations in blood and in coronary plaques of metabolic syndrome patients (Zorio et al., 2008)

## Leptin

### Obesity and Metabolic Dysfunction

Leptin is a polypeptide hormone produced by adipocytes and is considered a pro-inflammatory adipokine involved in the “low-grade inflammatory state” described in overweight and with obesity patients (Lago et al., 2009). It is well known that leptin is able to decrease food intake and increase energy expenditure by binding to and activating the long form of a membrane receptor (LEPR-B), highly expressed in the hypothalamus and other brain regions. The expression of LEPR-B was also detected in the nucleus of the solitary tract of the hindbrain, including subpopulations expressing glucagon-like peptide 1 (GLP-1) and cholecystokinin (CCK) (Rosenbaum and Leibel, 1999; Ahima et al., 2000; Schwartz et al., 2000; Myers et al., 2009).

The binding of leptin to LEPR-B leads to activation of a number of signaling pathways, such as Janus kinase 2 (JAK2)/signal transducer and activator of transcription 3 (STAT3), insulin receptor substrate (IRS)-1, phosphatidylinositol 3-kinase (PI3K), SH2-containing protein tyrosine phosphatase 2 (SHP2)-mitogen-activated protein kinase (MAPK), and 5’-adenosine monophosphate-activated protein kinase (AMPK)/acetyl-CoA carboxylase, and other pathways (Morris and Rui, 2009; Dalamaga et al., 2013; Moon et al., 2013; Park and Ahima, 2015). In particular, the activation of JAK2/STAT3 signaling was found to be critically involved in modulatory effects of leptin on energy homeostasis (Bates et al., 2003; Dardeno et al., 2010). The role of leptin in obesity-induced adipose inflammation has been described by Deng et al. (2013). In this context, leptin was found to stimulate CD4 + T_H_1 IFNγ secretion to induce adipocyte MHCII expression.

Leptin is known to be mainly involved in response and defense against reductions of body fat that could lead to impairment of survival and reproductive fitness. Therefore, CNS actions of leptin are necessary for energy stores, as well as normal energy homeostasis, reproduction, and the like.

Therefore, improving or restoring central leptin sensitivity is an interesting target in pharmacological treatment of weight loss.

The association of leptin with amylin, a peptide hormone released with insulin by pancreatic beta cells which modulate glucose and energy homeostasis, showed enthusiastic results, predominantly mediated by decreased feeding and increased central leptin sensitivity in diet-induced obesity (Roth et al., 2008; Trevaskis et al., 2010). In addition to its role in feeding control and energy expenditure, leptin also upregulates pro-inflammatory cytokines, including tumor necrosis factor (TNF)-α and interleukin (IL)-6; these factors are associated with insulin resistance and T2DM (López-Jaramillo et al., 2014). In this context, pro-opiomelanocortin (POMC)-expressing neurons located into the hypothalamic arcuate nucleus (ARC) are critically involved in leptin’s effects on glucose homeostasis in obesity and insulin resistance states (Huo et al., 2009; Berglund et al., 2012). In particular, leptin was found to activate anorexigenic neurons co-expressing POMC and cocaine- and amphetamine-regulated transcript (CART), and inhibit orexigenic neurons co-expressing agouti-related peptide (AgRP) and neuropeptide Y (NPY). During fasting, a rapid reduction of leptin concentrations in serum was found. The fall in leptin stimulates the expression of AgRP and NPY and suppresses POMC and CART, thereby resulting in stimulated food intake and reduced energy expenditure (Ahima et al., 1999; Cowley et al., 2001). Leptin was also found to inhibit neurons that express melanin-concentrating hormone and orexins, in the lateral hypothalamus, resulting in inhibition of food intake (Abizaid et al., 2006; Robertson et al., 2008). Regarding its role on energy expenditure, leptin has been reported to act primarily in the hypothalamus. The result of this signaling is an increase in the sympathetic tone that promotes lipid mobilization in the WAT and thermogenesis in the BAT through enhanced uncoupling protein 1 (UCP1) expression (Scarpace et al., 1997; Pandit et al., 2017).

Several studies showed that circulating levels of leptin are highly related to percentage of body fat in humans suffering from obesity and normal body weight individuals; increased size of adipocytes due to accumulation of triglyceride induces an increase of leptin as an adaptive response signaling control of energy balance to the central nervous system (CNS) (Rosenbaum and Leibel, 1999; Ahima et al., 2000; Schwartz et al., 2000; Hafizuallah, 2006; Myers et al., 2009). A more recent study on Saudi women with high body mass index (BMI), waist circumference, and metabolic syndrome confirmed a significant correlation between leptin levels and obesity (Al-Amodi et al., 2018). The first clinical trial that studied the potential pharmacological effects of leptin on polygenic or simple obesity dates back to 1999 (Heymsfield et al., 1999). In this context, recombinant methionyl human leptin was injected subcutaneous daily at high dose (e.g., a 100-kg patient in the 0.3 mg/kg dose group was administered 30 mg of leptin in 6 ml in two divided doses). The study, although original, was little appreciated because of inconsistent results and not requiring that patients reduced their food intake. On the other hand, recombinant leptin treatment is widely established and approved to treat obesity causes by leptin deficiency. Farooqi et al. (2002) showed that daily subcutaneous injections of recombinant human leptin for up to 4 years induced beneficial effects on various phenotypic abnormalities, such as appetite and fat mass, in children suffering from congenital obesity due to genetic deficiency of the leptin gene.

Over the years, different clinical trials have studied the potential effect of recombinant leptin to mitigate weight regain after caloric restriction or weight loss (Rosenbaum et al., 2005). Zelissen et al. (2005) evaluated the effects on body weight of subcutaneous administration of 10 mg recombinant leptin twice daily for 12 weeks associated with a restricted 500 kcal diet. However, this study did not show any significant change in body weight between active and placebo groups. Actually, elevated leptin levels in individuals affected by obesity point to a state of resistance to this peptide which could be due defects in leptin receptor signaling (Levin et al., 2004; Munzberg et al., 2004). Different studies on abnormal adipokine profiles in people suffering from obesity and T2DM have resulted in ambiguous findings. In these patients, Liu W. et al. (2020) described significantly increased leptin and reduced adiponectin levels. In addition, although it has been hypothesized a direct effect of leptin on pancreatic β-cell function (Lee et al., 2011), acting as an insulin sensitizer, and improving metabolism in patients with leptin-deficiency, it does not increase insulin sensitivity in humans with obesity (Mittendorfer et al., 2011) and does not lead to activation of intracellular signaling pathways and/or improve diabetes and glycemic control in individuals suffering from obesity and T2DM (Moon et al., 2011). Specifically, a short-term (2 weeks) pilot study conducted on humans suffering from obesity with T2DM treated with recombinant human leptin (20–80 mg/day) did not show improved insulin action, although recombinant leptin resulted in three- to 150-fold increase in plasma concentrations of leptin (Mittendorfer et al., 2011). In addition, in patients affected by obesity with T2DM, metreleptin injection at a dose of 10 mg/kg/day for 16 weeks did not modify body weight or circulating inflammatory markers but decreased HbA(1c) marginally (Moon et al., 2011).

Further studies are needed to clarify the role of leptin treatment in personalized management of people suffering from T2DM.

### Rheumatic Diseases

Leptin exerts modulatory effects on both innate and adaptive immunity activating LEPR expressed on immune cell, chondrocytes, and osteoblasts (Gómez et al., 2011; Conde et al., 2012; Procaccini et al., 2012). Different studies have described the involvement of the leptin in several physiological and pathophysiological conditions, including inflammation, immunity, and rheumatic diseases (Conde et al., 2014; Scotece et al., 2014b; Tian et al., 2014). A wide body of evidence showed that leptin serum levels are significantly increased in RA, and related to disease course and activity (Lee et al., 2007; Yoshino et al., 2011; Cao et al., 2016; Lee and Bae, 2016). In addition, the synovial/serum leptin ratio was positively associated with erosions in patients with RA compared to non-erosive RA controls (Olama et al., 2012). In particular, leptin was found to induce expression of IL-6 and IL-8 in synovial fibroblasts in patients with RA, via activation of JAK2/STAT3, nuclear factor kappa-light-chain-enhancer of activated B cells (NF-κB), and AP-1 signaling pathways (Muraoka et al., 2013; Yang et al., 2013), representing a new therapeutic target in the treatment of RA. On the other hand, cardiovascular risk is also highly related to obesity and RA pathology. Accordingly, Batun-Garrido et al. (2018) showed that leptin and IL-6 levels are associated with cardiovascular risk in RA patients (Batun-Garrido et al., 2018).

Leptin antagonists have been proposed for the prevention of RA in genetically susceptible individuals (Fazeli et al., 2006). In particular, a monoclonal antibody (mAb) against the human leptin receptor has been designed that blocks human monocyte and T cell activation in tissue-specific manner (Fazeli et al., 2006).

### Cardiovascular Diseases

There is growing evidence regarding the implication of leptin in the pathogenesis of CVD. Increased plasma leptin levels were observed in patients with essential hypertension (Beltowski, 2006; Momin et al., 2006; Katsiki et al., 2018). Leptin was also found to induce vasodilator effects in humans with coronary artery disease (Beltowski, 2006; Momin et al., 2006; Katsiki et al., 2018). However, administration of leptin did not modify blood pressure, probably because of concomitant stimulation of the sympathetic nervous system, natriuresis, and NO-dependent vasorelaxation (Beltowski, 2006). More recently, Simonds et al. (2014) reported that increased leptin levels in diet-induced obesity drive an increase in blood pressure in rodents, suggesting that pharmacological approaches based on the modulation of leptin effects on specific subpopulations of neurons could represent a potentially useful therapeutic strategy for obesity-associated hypertension and cardiovascular disease. In addition to its effects on blood pressure, leptin regulates cardiac contractile function, metabolism, cell size, and production of extracellular matrix (ECM) components in cardiomyocytes (Dong et al., 2006; Madani et al., 2006; Palanivel et al., 2006). In this context, Palanivel et al. (2006) reported the distinct effects of long- and short-term leptin treatment on glucose and fatty acid uptake and metabolism in HL-1 cardiomyocytes. These differences might lead to lipotoxic damage in heart failure (Palanivel et al., 2006). Moreover, leptin reduced myocardial reperfusion-induced death (Smith et al., 2006) and induced the elongation of myocardial cells and eccentric dilatation with compensation (Abe et al., 2007). In accordance with these results, it has been suggested an important role of leptin in the regulation of cardiomyocyte hypertrophy (Adiarto et al., 2007). As regards its effects on atherosclerosis, leptin was shown to play a critical role in the early phase of this disease. Leptin was able to initiate monocyte/macrophage recruitment into the vessel wall, increasing so the production of superoxides by mitochondria and expression of MCP-1 in aortic endothelial cells (Yamagishi et al., 2001). The inconsistent correlation of leptin levels with preclinical atherosclerosis may depend on the pathophysiological context of patients, medications, or other factors not yet identified. It can be said that therapeutic interventions targeting leptin might potentially prevent or reduce cardiovascular complications.

## Adiponectin

### Obesity and Metabolic Dysfunction

Adiponectin (also known as Acrp30, AdipoQ, GBP-28, and apM1) is a 244-amino acid protein se-creted by different cell types, such as adipocytes and endothelial cells (Scherer et al., 1995; Maeda et al., 1996; Achari and Jain, 2017). Different isoforms of adiponectin were described: globular, trimeric [low molecular weight (LMW)], hexameric [middle molecular weight (MMW)], and multimeric [high molecular weight (HMW)] adiponectin isoforms (Garaulet et al., 2007). The insulin-sensitizing and anti-inflammatory effects of adiponectin are mediated by two isoforms of adiponectin receptors (AdipoR1 and AdipoR2). In particular, AMPK phosphorylation and increased expression of peroxisome proliferator-activated receptor α (PPARα) through adiponectin receptor (AdipoR)1 and AdipoR2, respectively, are involved in mediating fatty acid metabolism and the insulin sensitizing actions of adiponectin in skeletal muscle (Kadowaki and Yamauchi, 2005; Ye and Scherer, 2013; Kim and Park, 2019). In this context, the binding of adiponectin with its receptors leads to the activation of various signaling pathways such as IRS-1/2, AMPK, and p38 MAPK. Activation of IRS-1/2 by adiponectin signaling is mainly involved in insulin-sensitizing effects of adiponectin in insulin responsive tissues. In addition, PI3K/Akt pathway plays a critical role in mediating metabolic actions of insulin, such as protein synthesis, lipogenesis, glucose uptake and utilization, glycogen synthesis, and reduced lipolysis and gluconeogenesis (Yamauchi et al., 2007).

In the liver, adiponectin activates glucose transporters and inhibits gluconeogenesis via AMPK, stimulating fatty acid oxidation and decreasing inflammation through the PPARα pathway (Wu et al., 2003). Adiponectin expression and release are strongly linked to healthy adipocyte differentiation, because they are modulated by PPARγ, and to insulin sensitivity. In fact, PPARγ was also found to regulate GLUT4 expression in adipocytes. In agreement, both expression and release of adiponectin are inhibited by inflammation and other stress phenomena occurring in obesity/T2DM (Astapova and Leff, 2012). Because of these positive actions, adiponectin has been widely investigated in human and animal models. In rodents, a significant increase of glucose uptake and fat oxidation in skeletal muscle, paralleled by reduced production of glucose in the liver and improved insulin sensitivity were found following administration of recombinant adiponectin (Berg et al., 2001; Fruebis et al., 2001; Yamauchi et al., 2001). Moreover, adiponectin was found to modulate feeding behavior and energy expenditure during fasting through central effects (Kubota et al., 2007). On the other hand, the central contribution of adiponectin is a rather marginal mechanism, being the main action in peripheral tissues.

It is well known the involvement of adiponectin in obesity-associated comorbidities, including T2DM and cardiovascular disease (Achari and Jain, 2017). Circulating levels of adiponectin are inversely associated with obesity, insulin sensitivity, T2DM, and metabolic syndrome (Arita et al., 1999; Hotta et al., 2000; Weyer et al., 2001; Mohan et al., 2005; Gilardini et al., 2006; Yamauchi et al., 2007; Wang et al., 2008).

### Rheumatic Diseases

Over the years, adiponectin has been mainly studied for its effects on metabolic syndrome, T2DM, and atherosclerosis. However, the opposing effects observed for some of its isorforms on effector cells of arthritis shifted the focus toward this disease (Neumann et al., 2011; Fatel et al., 2018).

In particular, HMW adiponectin was found to stimulate IL-6 secretion in human monocytes, while LMW adiponectin decreased IL-6 and stimulated secretion of IL-10 in LPS-activated monocytes (Neumeier et al., 2006). To evaluate the potential therapeutic effects of adiponectin in RA, Lee et al. (2018) generated various monoclonal antibodies (mAbs) specific to different adiponectin isoforms. Some clones of mAb, such as KH4-8, effectively inhibited expression of IL-6 and IL-8 in recombinant adiponectin-stimulated human osteoblasts, while other clones, KH7-33 and KH4-8, improved rheumatic symptoms in a mouse model of collagen-induced arthritis (CIA). Lee et al. (2018) found that antibodies against either MMW/HMW or MMW alone significantly ameliorated CIA in mice; therefore, a possible contribution of both adiponectin isoforms to progression of arthritis has been hypothesized. These results suggest that targeting adiponectin with mAb could be beneficial in inflammatory conditions. Previous studies showed a positive association between circulating adiponectin levels, disease activity, and radiographic disease progression (Neumann et al., 2011; Fatel et al., 2018). More recently, Liu R. et al. (2020) showed that intra-articular injection of adiponectin increased synovial inflammation by T follicular helper cells in a mouse model of CIA. Surprisingly, the correlation with disease activity has been found in some studies but not in others (Fatel et al., 2018), a discrepancy which still needs to be elucidated.

### Cardiovascular Diseases

Different reports have described the protective activity of adiponectin in atherosclerosis and cardiovascular disease (Ouchi and Walsh, 2007; Villarreal-Molina and Antuna-Puente, 2012).

Adiponectin decreased inflammation in vascular endothelial cells, smooth muscle cell, cardiac myocytes, and macrophages predominantly by activating AMPK and cyclic AMP-protein kinase A (cAMP-PKA) (Nigro et al., 2013; Subedi and Park, 2013). The antinflammatory activity of adiponectin may be mediated by direct effects on inflammatory cells or through suppression of the NF-kB signaling pathway (Ouchi et al., 2003; Kobayashi et al., 2004; Shibata et al., 2004; Nigro et al., 2013). In addition, studies showed that overexpression of adiponectin protects from atherosclerotic plaque formation (Okamoto et al., 2008), whereas deficiency of adiponectin results in increased incidence of atherosclerosis (Nawrocki et al., 2010). In particular, in adiponectin knocked out mice, there have been observed inflammatory vascular lesions following mechanical injury, with neointimal thickening and stimulated proliferation of vascular smooth muscle cells that was shown to be attenuated by restoring physiological levels of adiponectin (Kadowaki and Yamauchi, 2005). The vasoprotective effects of adiponectin could be due to increased production of nitric oxide through activation of endothelial NO synthase (eNOS) via AMPK-dependent (Kobayashi et al., 2004; Ouchi et al., 2004) as well as cyclooxygenase 2-dependent mechanisms (Ohashi et al., 2009).

Recently, plasma concentrations of adiponectin have been investigated in patients with coronary artery disease and slow coronary flow. Low plasma levels of adiponectin were found in atherosclerotic coronary artery disease (Diah et al., 2019). A recent metanalysis showed that pravastatin, a 3-hydroxy-3-methylglutaryl coenzyme reductase inhibitor, widely used in the primary and secondary prevention of cardiovascular diseases, increases circulating adiponectin levels, which could be a mechanism underlying the beneficial effects of pravastatin in atherosclerosis (Shu and Chi, 2019).

However, some studies have suggested that the association between adiponectin concentrations and risk of vascular events is strictly dependent on ethnicity (Gardener et al., 2013). Therefore, it is necessary to obtain more data in diverse populations.

## Resistin

### Obesity and Metabolic Dysfunction

Resistin (or “resistance to insulin”) was discovered in mice in 2001 as an adipocyte-derived signaling molecule, and named for its role in inducing insulin resistance (Steppan et al., 2001a). Resistin belongs to a family of resistin-like molecules which include various members with distinct expression patterns and biological effects (Steppan et al., 2001b). Human resistin is expressed in adipose tissue, bone marrow, lung (Patel et al., 2003), placental tissue (Yura et al., 2003), pancreatic islet cells (Minn et al., 2003), and plasma (Yannakoulia et al., 2003). Inconsistent associations between serum resistin levels and body fat have been found with positive (Zhang et al., 2002), inverse (Way et al., 2001; Rajala et al., 2002; Steppan and Lazar, 2002) or no correlations (Fain et al., 2003), in rodents. Increased serum resistin levels were found in humans suffering from obesity respect to lean individuals (Schäffler et al., 2004; Vendrell et al., 2004). Moreover, elevated resistin levels were observed to induce insulin resistance in several rat and mouse models (Rajala et al., 2004; Rangwala et al., 2004; Satoh et al., 2014), suggesting an involvement of hyper-resistinemia in insulin resistance. However, the possible relationships between circulating resistin levels and insulin resistance in individuals affected by obesity with and without diabetes have not been clarified. Al-Harithy and Al-Ghamdi (2005) found a positive correlation between serum resistin levels and insulin resistance in women with T2DM and women suffering from obesity or overweight women whithout T2DM, while other studies failed to report associations between serum resistin levels and insulin resistance (Gerber et al., 2005; Bu et al., 2012). The discrepant results were probably due to small sample sizes, population differences, disease status, and use of non-standardized assays of resistin (Huang and Yang, 2016; Park H. K. et al., 2017). Recently, a metanalysis of 15 studies confirmed a positive correlation between circulating resistin levels and insulin resistance in individuals suffering from obesity and T2DM with hyperresistinemia, but not in patients with normal resistin levels. In this context, resistin has been hypothesized as a new therapeutic target for the prevention and treatment of diabetes mellitus (Su et al., 2019).

### Rheumatic Diseases

The role of resistin in regulating inflammatory pathways in the context of rheumatic diseases has been well verified. Resistin expression was detected in synovial fluid (SF) and serum of both RA and OA patients. In particular, resistin was shown to accumulate in the inflamed joints of RA patients and to induce the development of arthritis accompanied by leukocytic infiltration and hyperplasia of the synovium following injection into the joints of healthy mice. In addition, different reports suggest that resistin has pro-inflammatory properties (Bokarewa et al., 2005). Interestingly, Kassem et al. (2010) reported that serum resistin could be a good prognostic marker in RA. Thommesen et al. (2006) showed that resistin exerts stimulatory effects on osteoblast proliferation and its expression is increased during osteoclast differentiation, through protein kinase C (PKC) and PKA signaling mechanisms. In human articular chondrocytes, resistin treatment led to increased expression of various cytokines and chemokines, including TNF-α, IL-6, and IL-12 (Zhang Z. et al., 2010), along with different catabolic enzymes and markers of cartilage, including metalloproteinase (MMP)-1, MMP-2, and ADAMTS-4, while it decreased type II collagen alpha 1 and aggrecan (Fang et al., 2011).

Recently, resistin was also found to promote expression of MCP-1 in synovial fibroblasts from OA patients and monocyte migration (Chen et al., 2019), confirming potential therapeutic implications of this adipokine for patients affected by OA.

### Cardiovascular Diseases

A positive correlation between serum resistin levels and hypertension was shown in humans (Takata et al., 2008; Zhang L. et al., 2010; Thomopoulos et al., 2011). In this context, the potential role of resistin as a risk factor for hypertension has also been confirmed in a recent meta-analysis (Zhang et al., 2017). Moreover, resistin has been suggested to play a modulatory role on angiogenesis, thrombosis, and vascular smooth muscle cell migration and proliferation, which contribute to pathogenesis and progression of atherosclerosis (Jamaluddin et al., 2013). In particular, human resistin was able to cause a significant increase in the expression of pro-inflammatory factors, such as MCP-1, endothelin-1, and MMPs (Manduteanu et al., 2010; Hsu et al., 2011). Human resistin was also found to increase the proliferation and migration of human endothelial cells and vascular smooth muscle cells and endothelial permeability, through oxidative stress and activation of p38 MAPK signaling pathways (Jamaluddin et al., 2013). Associations between serum resistin levels and development of coronary artery disease were also observed in humans. A number of studies found increased levels of resistin in angina pectoris patients undergoing coronary angiography (Lee and Kim, 2012). Elevated resistin levels were found to be predictive of coronary atherosclerosis, independently of CRP levels, in asymptomatic subjects (Park and Ahima, 2013). Resistin is also a predictor of major adverse cardiovascular events in patients with coronary artery disease, including cardiovascular death and myocardial infarction (MI; On et al., 2007; Kreçki et al., 2011). Additionally, various studies confirmed elevated levels of resistin in humans with acute coronary syndromes and its relationship with severe myocardial injury and poor prognosis (Lee and Kim, 2012). On the other hand, further studies are necessary to understand the specific signaling mechanisms involved in the effects of resistin in cardiovascular disease.

## Chemerin

### Obesity and Metabolic Dysfunction

Chemerin is an inflammatory cell-derived chemokine structurally and evolutionary related to cathelicidin precursors (antibacterial peptides), cystatins (cysteine protease inhibitors), and kininogens (Wittamer et al., 2003). It is highly expressed in adipose tissue *in vivo* and showed to promote adipogenesis in a 3T3-L1 fibroblast preadipocyte model (Goralski et al., 2007). Different chemerin isoforms exist depending on protease mediated cleavage of the peptide sequence (Zabel et al., 2005; Yamaguchi et al., 2011; Zhao et al., 2018). Wittamer et al. (2004) showed that cleavage must be extremely specific in order to maintain the active chemerin S157 peptide as an agonist on its G-protein-coupled receptor encoded by the gene CMKLR1 (also known as ChemR23). ChemR23 is widely known to act through Gi and ERK1/2 (Wittamer et al., 2003); however, Kaur et al. (2010) demonstrated that chemerin-induced endothelial angiogenesis is mediated through ChemR23 and downstream activation of Akt (PKB), p38, and ERK1/2 (Kaur et al., 2010). The connection between chemerin and obesity indicates that it may contribute to the development of obesity. Elevated chemerin gene expression and its receptor were observed in adipose tissue of obese and type 2 diabetic gerbil *Psammomys obesus* with respect to lean, normoglycemic *P. obesus* (Bozaoglu et al., 2007). On the other hand, food restriction was accompanied by reduced gene expression of chemerin in WAT, in rats (Stelmanska et al., 2013). In addition, chemerin administration displayed modulatory effects on several adipokines such as leptin, adiponectin, and IL-6, which are involved in the modulation of metabolic and inflammatory responses (Goralski et al., 2007). In humans, the existence of a positive correlation was found between both local and circulating levels of chemerin, BMI, and obesity-related biomarkers. Moreover, chemerin has been hypothesized as a possible link between obesity and the development of T2DM (Ernst and Sinal, 2010). Gene expression of chemerin was increased in adipose tissue of patients suffering from obesity with T2DM (Bozaoglu et al., 2007). In mouse preadipocyte cell line 3T3-L1, chemerin has been suggested to stimulate insulin sensitivity in adipose tissue by enhancing insulin-stimulated uptake of glucose and IRS-1 tyrosine phosphorylation (Kalra et al., 1999; Seeger et al., 2008). In humans, no significant differences in plasma chemerin concentrations were found between T2DM patients and normal subjects. On the other hand, in subjects with normal glucose tolerance, circulating levels of chemerin are reported to be significantly associated with BMI, circulating triglycerides and blood pressure (Bozaoglu et al., 2007). These results indicate that chemerin plays a pivotal role in the modulation of energy balance, making it a promising pharmacological tool in obesity treatment.

### Rheumatic Diseases

Different studies described a positive correlation between systemic chemerin and CRP in various chronic inflammatory diseases, including rheumatic diseases (Buechler, 2014; Fatima et al., 2014; Mariani and Roncucci, 2015). In agreement, chemerin might have a pivotal role in the pathophysiology of RA (Buechler, 2014; Fatima et al., 2014; Mariani and Roncucci, 2015). Patients with rheumatic diseases express both chemerin and its receptor in chondrocytes and synovial fibroblasts (Berg et al., 2010; Kaneko et al., 2011; Yamaguchi et al., 2011). However, the biological effects of its different isoforms in rheumatic diseases are not clearly defined. Mouse chem156S, homologous to human chemerin isoform chem157S, showed antinflammatory activity in macrophage (Cash et al., 2008) and in LPS-induced acute lung injury model (Luangsay et al., 2009). Moreover, Zhao et al. (2018) described the isoform chem156F in SF samples from patients with rheumatic diseases. Chemerin has been hypothesized to stimulate synovial fibroblasts motility and leukocyte migration into the joint, as well as the expression of IL-6, MCP1, MMP-3, and Toll-like receptor 4 (TLR4) in synovial fibroblasts from RA patients (Conde et al., 2011; Kaneko et al., 2011; Eisinger et al., 2012). Chemerin could therefore play a critical role in innate and adaptative immunity by acting as a chemoattractant agent for natural killer cells, macrophages, and dendritic cells (Meder et al., 2003; Wittamer et al., 2003). Interestingly, tocilizumab, a humanized mAb directed to IL-6 receptor, decreased serum chemerin levels in RA patients (Fioravanti et al., 2019).

### Cardiovascular Diseases

Besides its role in adipogenesis and energy metabolism (Bozaoglu et al., 2007; Goralski et al., 2007; Ernst et al., 2010; Roman et al., 2012), recent studies reported a key role for chemerin in the pathologenesis of cardiovascular diseases, including coronary heart disease (Kaur et al., 2018). Recently, Wu et al. (2020) reported increased epicardial fat volume, lower adiponectin expression and higher chemerin and vascular endothelial growth factor (VEGF) expression in patients with coronary artery disease compared to healthy individuals. Despite some limitations, the study reported that expression of adiponectin gradually decreased with the increase of epicardial fat volume, while the expression of chemerin and VEGF gradually increased with the increase of epicardial fat volume, displaying a positive correlation with coronary artery disease. In addition, a study conducted in 40 patients suffering from T2DM with cardiovascular disease showed that serum concentrations of chemerin, omentin-1, and visfatin correlated with the prognosis of cardiovascular complications. In particular, chemerin and visfatin (pro-inflammatory markers) levels were positively correlated with increased C-reactive protein (CRP), triglycerides, fasting blood glucose (FBG), serum cholesterol, and microalbuminuria, while omentin-1 (antinflammatory marker) levels were significantly reduced (Ahmed et al., 2020). On the other hand, chemerin stimulates the progression of atherosclerosis in ApoE–/– mice, confirming its crucial role in the pathologic process of cardiovascular diseases (Liu et al., 2019). In addition, atorvastatin and rosuvastatin were found to decrease chemerin levels in patients with acute MI (Tunçez et al., 2019). On the basis of these findings, it has been suggested a significant role of chemerin in human vascular health and disease.

## LCN2

### Obesity and Metabolic Dysfunction

Lipocalin 2 (LCN2), also known as neutrophil gelatinase-associated lipocalin (NGAL), is a glycoprotein recently identified as a cytokine derived from adipose tissue. It is able to bind small substances, including steroids and lipopolysaccharides (Flower, 1996; Chu et al., 1998). Expression of LCN2 was shown in a number of tissues such as uterus (Huang et al., 1999), immune cells (Borregaard and Cowland, 2006), liver, spleen, kidney, and bone marrow (Cowland and Borregaard, 1997; Aigner et al., 2007). Two receptors have been identified for LCN2: the solute carrier family 22 member 17 (SLC22A17 or 24p3R) binding mouse LCN2 (Devireddy et al., 2005) and the megalin/glycoprotein GP330, a low-density lipoprotein receptor involved in binding of human LCN2 protein (Hvidberg et al., 2005). Different studies reported its role in apoptosis (Devireddy et al., 2001), transport of fatty acids (Chu et al., 1998) and iron (Yang et al., 2002), regulation of inflammation (Cowland and Borregaard, 1997), and metabolism (Yan et al., 2007).

Despite LCN2 expression is altered in obesity (Sommer et al., 2009; Jang et al., 2012), its role in the pathogenesis of this disease is not entirely clear. Transcriptional activation of LCN2 gene in adipose tissue has been hypothesized to be related to inflammation and obesity (Garay-Rojas et al., 1996; Shen et al., 2006). Notably, a role for LCN2 in the adipose tissue remodeling has also been suggested (Garay-Rojas et al., 1996; Shen et al., 2006). On the other hand, Xiaoli Chen’s group demonstrated that molecular disruption of LCN2 in mice significantly exacerbates diet-induced obesity, dyslipidemia, fatty liver disease, and insulin resistance (Guo et al., 2010). In this context, LCN2 may be considered one of the mediators responsible for the low-level systemic inflammation observed in metabolic syndrome associated with obesity (Jang et al., 2012; Abella et al., 2015), representing a future therapeutic target.

### Rheumatic Diseases

In joint tissues, LCN2 expression is induced after stimulation with inflammatory factors and in response to mechanical loading (Gupta et al., 2007; Katano et al., 2009; Conde et al., 2011). Elevated LCN2 concentrations were found in SF of RA patients (Gupta et al., 2007; Katano et al., 2009; Staikos et al., 2013). LCN2 is also believed to play a role in degenerative articular diseases (Gómez et al., 2011), and it has been suggested as a biomarker of cartilage degradation in arthritic diseases (Wilson et al., 2008). In chondrocytes, IL-1β, LPS, certain adipokines (leptin and adiponectin), and dexamethasone were found able to modulate LCN2 expression (Conde et al., 2011). Moreover, it was also suggested to establish a feedback regulatory loop with nitric oxide (Gomez et al., 2013). Although some data suggest that LCN2 is involved in joint pathophysiology, further studies are needed to better clarify its role in clinical rheumatology.

### Cardiovascular Diseases

Increasing evidences suggest the involvement of LCN2 in inflammatory processes in cardiovascular diseases (Sivalingam et al., 2017). In particular, LCN2 plays a crucial role in vascular remodeling and plaque instability in atherosclerosis (Hemdahl et al., 2006; Folkesson et al., 2007). LCN2 has been reported to regulate the enzymatic activity of MMP-9, acting as a key mediator of plaque instability in atherosclerosis (Cakirca and Turgut, 2018). In addition, elevated LCN2 concentrations have been found in heart failure, coronary heart disease, and stroke (Elneihoum et al., 1996; Damman et al., 2008; Yndestad et al., 2009; Zografos et al., 2009), providing biological plausibility for the potential role of LCN2 as a biomarker in cardiovascular disease. Despite a wide literature supporting a role of LCN2 in the pathophysiology of cardiovascular disease, to date there are insufficient data regarding the clinical usefulness of monitoring LCN2 concentrations in the treatment of cardiovascular diseases (Cruz et al., 2012).

## Vaspin

### Obesity and Metabolic Dysfunction

Visceral adipose tissue-derived serpin (vaspin) is a 47-kDa protein, belonging to the serine protease inhibitor family (Hida et al., 2000). Human, rat, and mouse vaspin proteins are constituted by 395, 392, and 394 amino acids, respectively, and display a 40% homology with α_1_-antitrypsin (Hida et al., 2005). Increasing numbers of studies emphasize the role of vaspin in obesity and related metabolic disorders. Vaspin was found to be mainly expressed in visceral adipose tissue of Otsuka Long-Evans Tokushima fatty (OLETF) rats (Hida et al., 2000, 2005), an animal model of T2DM characterized by abdominal obesity, insulin resistance, hypertension, and dyslipidemia (Kawano et al., 1992), with higher plasma levels at age 30 weeks, concomitantly with peak of insulin concentrations. In humans, vaspin was also reported to be expressed in several tissues, such as visceral and subcutaneous adipose tissue and skeletal muscle (Klöting et al., 2006; Klöting et al., 2011; Nicholson et al., 2018, 2019). Interestingly, human vaspin mRNA expression in adipose tissue was found to be modulated in a fat depot-specific manner and to be related to obesity and insulin resistance (Klöting et al., 2006). In addition, vaspin was more expressed in subcutaneous adipose tissue and skeletal muscle of older patients with obesity compared to lean age-matched controls (Nicholson et al., 2019). A recent metanalysis of six studies aimed to evaluate the relationship between vaspin and obesity confirmed increased concentrations of vaspin in patients suffering from obesity and T2DM (Feng et al., 2014). Administration of recombinant human vaspin to obese mice fed a high-fat and high-sucrose diet was found to improve glucose tolerance and insulin sensitivity, reduce the gene expression of leptin, TNF-α, and resistin, and stimulate adiponectin gene expression (Hida et al., 2005), suggesting an insulin-sensitizing role for vaspin. Klöting et al. (2011) showed that central and peripheral vaspin administration to both obese db/db and lean C57BL/6 mice led to a reduction in food intake. More recently, Heiker et al. (2013) found that recombinant vaspin administration induced a similar increase in insulin sensitivity in both db/db and C57BL6 mice. The same authors proposed that the anorexigenic effects of vaspin could be related to inhibition of a protease that degrades a putative anti-orexigenic factor. Similarly, we have previously reported that administration of vaspin into the hypothalamic ARC of rats decreased food intake, probably due to reduction of NPY and increase of POMC mRNA levels (Brunetti et al., 2011). In humans, serum vaspin concentrations displayed a pre-prandial rise followed by a significant reduction in response to meals (Jeong et al., 2010). Moreover, vaspin serum concentrations have been reported to be significantly related to obesity and impaired insulin sensitivity. However, this correlation seemed to be abrogated in T2DM (Youn et al., 2008). It has been proposed that the effects of vaspin on glycemic control are mediated by kallikrein 7, a protease cleaving human insulin *in vitro*. Vaspin was found to inhibit kallikrein 7 through its serpin activity. Therefore, vaspin-induced antidiabetic effects could be related, at least in part, to a decreased degradation of circulating insulin (Heiker et al., 2013). In addition, vaspin has been suggested to be one of the effector molecules under the control of PPARγ, as confirmed by upregulated vaspin mRNA expression in rodents following pioglitazone treatment (Wada, 2008). Additional data in humans will facilitate the entire understanding of vaspin mechanism of action, making it possible to develop novel treatment strategies.

### Rheumatic Diseases

The potential role of vaspin in rheumatic diseases, including OA and RA, has not been fully evaluated. Vaspin was found to attenuate receptor activator of nuclear factor kappa-B ligand (RANKL)-induced osteoclast formation in murine macrophage cell line RAW264.7, decrease apoptosis of human osteoblasts, and regulate the osteogenic differentiation of pre-osteoblast cell line MC3T3-E1 (Kamio et al., 2013; Liu et al., 2016). Recent *in vitro* studies demonstrated that vaspin exerted protective effects against high fat diet-induced bone loss, and promoted osteoblastic differentiation through activation of the Smad-Runx2 signaling pathway (Wang et al., 2020). Conflicting reports were published on serum vaspin levels in rheumatic diseases. In particular, Bao et al. (2014) showed that vaspin serum concentrations were lower in OA patients respect to healthy subjects. On the other hand, higher serum vaspin levels were found in psoriatic arthritis and RA patients respect to healthy subjects (Ozgen et al., 2010; Colak et al., 2019). Importantly, Klaasen et al. (2012) showed that vaspin levels were increased following treatment with glucocorticoids in RA patients. In other works, no association was found between serum vaspin levels and serum CRP levels or leukocyte count in SF of RA patients (Senolt et al., 2010). In an opposite way, vaspin serum levels did not show statistically significant differences in juvenile idiopathic arthritis children with active joints compared to patients with no active joints and no association was found between plasma vaspin levels and presence or number of active joints (Cantarini et al., 2011).

### Cardiovascular Diseases

Limited and inconsistent clinical data are available regarding the association between vaspin levels and cardiovascular disease. Serum vaspin levels were significantly decreased in patients with coronary artery disease respect to healthy controls (Kadoglou et al., 2011). Similarly, decreased serum vaspin concentrations were observed in patients suffering from coronary artery disease, with respect to age-matched normal subjects (Kobat et al., 2012). In contrast, a positive association of serum vaspin levels with carotid atherosclerosis was found, independently of insulin resistance, in the general population (Esaki et al., 2014). Recently, Hao et al. (2016) reported that concentrations of vaspin were associated with the presence of both coronary artery disease and T2DM. A more recent report showed that vaspin cannot be considered as a biochemical marker for diagnosis of coronary artery disease in the general population (Stančík et al., 2017). In contrast, recently vaspin has been found highly expressed in human macrophages and vascular smooth muscle cells in coronary atheromatous plaques (Sato et al., 2018). As for the potential mechanisms mediating the effects of vaspin in vascular endothelial cells, it has been hypothesized that this peptide might protect blood vessels through prevention of free fatty acid-induced apoptosis in human vascular endothelial cells via upregulation of the PI3K/Akt/eNOS signaling pathway (Jung et al., 2011; Zieger et al., 2018). Vaspin was found to inhibit ICAM-1 expression induced by TNF-α by preventing generation of reactive oxygen species and activating NF-κB/PKC theta within vascular smooth muscle cells (Phalitakul et al., 2011; Liu et al., 2014). More recently, vaspin was found to suppress migration and proliferation of vascular smooth muscle cells, through downregulating ERK1/2 and c-Jun N-terminal kinase (JNK) pathways, and increasing collagen production through upregulation of PI3K/Akt pathways (Sato et al., 2018). Unfortunately, further studies are needed in order to elucidate the role of vaspin as a biomarker and/or a therapeutic agent in atherosclerotic cardiovascular disease.

## Omentin

### Obesity and Metabolic Dysfunction

Omentin-1 and omentin-2 are fat depot specific secretory proteins and their gene expression was reported in visceral stromal vascular cells, but not fat cells (Yang et al., 2006; Rabe et al., 2008). In humans, omentin-1 and omentin-2 genes are located adjacent to each other in the 1q22–q23 chromosomal region, which is linked to T2DM in several populations (de Souza Batista et al., 2007). Omentin-1 is the most common isoform in human plasma (de Souza Batista et al., 2007). Expression of this isoform is predominantly found in human omental adipose tissue specifically in visceral but not subcutaneous tissue, slightly expressed in gut, lung, heart, and rarely reported in muscle and kidney (Fu et al., 2004). Omentin-1 showed antinflammatory properties and has been identified as a major visceral (omental) adipokine which plays important roles in glucose homeostasis, lipid metabolism, insulin resistance, and diabetes (Senthilkumar et al., 2018). Omentin increases insulin signal transduction through activation of Akt/PKB, modulating body fat distribution between visceral and subcutaneous fat depots (Yang et al., 2006).

However, an inverse correlation between serum levels of omentin-1 and obesity was reported (de Souza Batista et al., 2007). Omentin enhances the effect of insulin on glucose metabolism (de Souza Batista et al., 2007) and its circulating concentrations are elevated after weight loss (Moreno-Navarrete et al., 2010). Furthermore, omentin levels are positively correlated with adiponectin and high-density lipoprotein levels (de Souza Batista et al., 2007). Howewer, further studies are needed to define the physiological role of omentin in glucose metabolism, its target tissues, receptors, or relevant signal transduction pathways.

### Rheumatic Diseases

Although a positive relationship between excessive weight and increased risk of developing RA in autoantibody positive individuals has been suggested, probably by production of various bioactive peptides, including omentin, it is not entirely clear the role of this peptide in rheumatic diseases. In an explorative study, conducted by Maijer et al. (2015), it has been reported a positive association between serum omentin and increased inflammatory state in autoantibody positive subjects at risk of developing RA. On the other hand, a reduction of omentin-1 concentrations in SF omentin-1 levels was observed in chronic inflammatory RA with respects to OA patients (Senolt et al., 2010), and SF levels were negatively correlated with self-reported pain and physical disability in OA patients (Li Z. G. et al., 2012). An independent relationship was showed between omentin and MMP-3 levels in patients with mild but not severe RA (Robinson et al., 2017). To date, the role of omentin in rheumatic diseases is unclear.

### Cardiovascular Diseases

It is well known that omentin-1 plays a pivotal role in vasodilatation, development of endothelial dysfunction, and arterial calcification (Tsuji et al., 2001; Yamawaki et al., 2010, 2011; Duan et al., 2011). Similarly to adiponectin, omentin-1 induces the activation of AMPK and endothelial nitric oxide synthase (Chen et al., 2003; Stejskal et al., 2003), playing a role in cellular energy homeostasis and vascular tone regulation. In addition, omentin-1 was found to be able to inhibit TNF-α production in vascular endothelial cells (Tan et al., 2010). In particular, omentin exerts a NO-mediated endothelium-dependent vasodilating effect in rat isolated vessels, *in vitro* (Yamawaki et al., 2010), probably related, at least in part, to a direct phosphorylation of endothelial nitric oxide synthase (Yamawaki et al., 2010). Omentin was also reported to induce revascularization and endothelial cell function through the stimulation of the Akt/eNOS signaling pathway (Maruyama et al., 2012). Antinflammatory effects of omentin, including suppression of inflammation induced by TNF-α and CRP in vascular endothelial cells, have been proposed to be involved in its vasoprotective effects, in humans (Tan et al., 2010; Yamawaki et al., 2011), and reduced concentrations of omentin in plasma have been proposed as a marker of endothelial dysfunction (Moreno-Navarrete et al., 2011). In addition, it has been observed that chronic omentin administration suppresses monocrotaline-induced pulmonary arterial hypertension in rats (Kazama et al., 2013). Moreover, our research group showed that subacute omentin-1 administration significantly decreased blood pressure and pulse pressure, in normotensive rats, probably through enhanced synthesis of NO in the vascular system, which, in turn, could be mediated by stimulated adiponectin and inhibited IL-6 gene expression in pericardial adipose tissue (Brunetti et al., 2014). Omentin-1 concentrations are inversely associated with markers of obesity, suggesting a possible role for omentin-1 in obesity related cardiovascular disease (de Souza Batista et al., 2007).

## FSTL1

### Obesity and Metabolic Dysfunction

Follistatin-like 1 was first described in 1993 as TSC-36 (TGFB-stimulated clone 36) in a study designed to identify genes regulated by transforming growth factor beta (Shibanuma et al., 1993). Thereafter, it was reported as a preadipocyte/adipocyte-secreted protein with a possible implication in obesity and inflammation (Lehr et al., 2012; Fan et al., 2013). Overweight and mild obesity might be associated with increased FSTL1 levels; however, Horak et al. (2018) observed a significant decline in FSTL1 level in individuals suffering from severe obesity compared to non-obese subjects. The authors suggested that decreased FSTL1 concentrations observed in people with severe obesity result from a reduction of adipogenesis accompanied by a senescence of preadipocytes, increased adipocyte apoptosis, and epigenetic FSTL1 silencing (Horak et al., 2018). Furthermore, FSTL1 exerted pro-inflammatory effects in both 3T3-L1 adipocytes and RAW264.7 macrophages. In particular, recombinant FSTL1 increased mRNA levels of IL-6, TNF-α, and MCP-1, in a dose-dependent manner, and activated IKKβ-NFκB and JNK signaling pathways in both cell lines. In addition, the same authors demonstrated an impairment of insulin signaling induced by FSTL1, in 3T3-L1 adipocytes, as confirmed by the attenuation of phosphorylation of both Akt and IRS-1 following insulin treatment. These findings suggest a possible role for FSTL1 as a mediator of inflammatory response and insulin resistance in obesity (Fan et al., 2013).

### Rheumatic Diseases

Controversial data have been reported for FSTL1 with respect to its possible effects on inflammation in rheumatic diseases. In CIA model, FSTL1 promoted inflammation, while in anti-type II collagen antibody-induced arthritis (CAIA) model, it inhibited inflammation. Opposite results might due to differences in disease development in animal models or in the extensive post-transcriptional regulation of FSTL1 (Mattiotti et al., 2018). Interestingly, FSTL1 has been shown to trigger inflammatory responses in both immune and non-immune cells, inducing TNF-α, IL-1β, and IL-6 secretion, in *in vitro* studies (Miyamae et al., 2006). Moreover, recombinant FSTL1 stimulated secretion of IFN-γ by T cells (Clutter et al., 2009), and the pro-inflammatory actions of FSTL1 have been suggested to be mediated by activation of TLR4 signaling (Murakami et al., 2012). *In vivo* studies demonstrated a causative role of FSTL1 in inflammation (Miyamae et al., 2006; Clutter et al., 2009). Accordingly, adenovirus-mediated administration of FSTL1 to mice increased CIA (Miyamae et al., 2006), while its neutralization with specific antibody showed antinflammatory effects (Clutter et al., 2009).

In addition, significantly increased levels of FSTL1 in serum and its overexpression in synovial tissue were observed in patients with RA (Clutter et al., 2009; Li et al., 2011). However, FSTL1 overexpression could be a consequence of RA rather than its cause (Ehara et al., 2004).

### Cardiovascular Diseases

Follistatin-like 1 has been reported to participate in modulating a number of biological processes, such as vascularization and immunomodulation and signaling pathways (Mattiotti et al., 2018). Its cardioprotective role has been intensively studied, though its mechanisms remain unclear. In multiple models of myocardial injury, the overexpression of FSTL1 has been demonstrated to act as a protective factor (Oshima et al., 2008). On the other hand, FSTL1 KO neonatal mice showed an overall enlargement of the heart (Sylva et al., 2011). In addition, decreased ischemic damage and improved cardiac performance after reperfusion were found following treatment with FSTL1, in preclinical model (Ogura et al., 2012). Intramuscular administration of an adenoviral vector expressing FSTL1 (Ad-FSTL1) induced a significant elevation of perfusion recovery and capillary vessel formation in the ischemic hind limbs of mice. In addition, Ad-FSTL1 enhanced migration and differentiation into vascular-like structures and suppressed apoptosis under conditions of serum deprivation, in cultured endothelial cells (Ouchi et al., 2008). The stimulatory effects of FSTL1 on endothelial cell function and revascularization following ischemic insult were associated with phosphorylation of Akt and stimulation of eNOS (Ouchi et al., 2008). A study of cardiac regeneration showed that FSTL1 induced various effects on cardiomyocyte proliferation and protection from apoptosis related to post-translational modification of the protein (Wei et al., 2015). However, patients with heart failure and acute coronary syndrome showed a significant increase of FSTL1 circulating concentrations (Widera et al., 2009; El-Armouche et al., 2011; Sylva et al., 2011; Tanaka et al., 2016). In this context, FSTL1 serum levels were correlated with mortality (Widera et al., 2009). Consequently, FSTL1 could have a negative prognostic value in these patients (El-Armouche et al., 2011; Widera et al., 2012).

## SPARC

### Obesity and Metabolic Dysfunction

Secreted protein acidic and rich in cysteine, also known as osteonectin or BM40, is a profibrotic protein that seems to have pleiotropic functions (Termine et al., 1981; Kos and Wilding, 2010). It exerts various functions, including modulation of the assembly and organization of ECM (Bornstein, 1995; Bradshaw, 2009), and regulation of a number of intracellular signaling pathways, in addition to affecting cell migration, proliferation, and differentiation (Chlenski and Cohn, 2010). In particular, it has been reported to bind various structural and soluble ECM proteins in a Ca^2+^-dependent manner, such as collagens (Sage et al., 1989; Sasaki et al., 1997; Chlenski et al., 2004; Kaufmann et al., 2004), vitronectin (Rosenblatt et al., 1997), fibrinogen fragments (Wang et al., 2006), thrombospondin-1 (Clezardin et al., 1988, 1991), VEGF (Kupprion et al., 1998), and platelet-derived growth factor (PDGF) (Raines et al., 1992). Although the functional role of this binding is not completely clear, SPARC has been proposed as an extracellular chaperone (Martinek et al., 2007). It was found that SPARC is secreted by adipocytes, inhibited adipogenesis, and promoted adipose tissue fibrosis (Kos et al., 2009; Lee et al., 2009; Kos and Wilding, 2010). In addition, studies suggested the involvement of SPARC in the pathogenesis of obesity and T2DM (Termine et al., 1981). Overexpression of SPARC modulates the expression levels of various pro-inflammatory cytokines, critically involved in insulin resistance, glucose and lipid metabolism during adipogenesis (Shen et al., 2014). Moreover, SPARC regulated thermogenesis in white and brown adipocytes, directly interacting with VEGF in adipocytes (Mukherjee et al., 2020). Recently, deletion of SPARC expression has been reported to cause diabetes mellitus in mice, demonstrating that SPARC deficient mice represent a reliable model for obesity and its metabolic complications, including diabetes mellitus (Atorrasagasti et al., 2019). On the other hand, clinical studies showed a link between increased SPARC levels and T2DM, diabetic retinopathy, and nephropathy (Munjal et al., 1994; Kanauchi et al., 2000; Taneda et al., 2003). A cross-sectional study on women with gestational diabetes mellitus showed higher SPARC and lower adiponectin levels as compared to normal glucose tolerance controls. Importantly, SPARC levels significantly correlated with inflammation and dyslipidemia, pointing to a potential involvement of SPARC in the pathophysiology of gestational diabetes mellitus (Xu et al., 2013). Approximately 20% of children with obesity exhibited the metabolically healthy obese (MHO) phenotype. A more favorable adipokine profile, characterized by decreased osteonectin, leptin, and RBP-4 and increased adiponectin levels was shown in MHO children with respect to metabolically unhealthy children suffering from obesity. In addition, higher levels of leptin, resistin, and RBP-4 paralleled by lower adiponectin, but similar osteonectin and FGF-21 levels, were reported in MHO children as compared to normal weight healthy controls. Therefore, a possible involvement of dysregulation of adipokines in the metabolic consequences of obesity in children can be suggested. It has been proposed that reduced concentrations of SPARC, paralleled by a healthy adipokine profile, might be used as an early marker of the MHO phenotype (Fu et al., 2018). Therefore, SPARC might represent a key player in the pathology of obesity and metabolic syndrome.

### Rheumatic Diseases

Secreted protein acidic and rich in cysteine could also be implicated in the pathogenesis of rheumatic diseases. Although widely expressed in mammalian tissues, the protein SPARC represents the major non-collagenous protein in bone (Bradshaw and Sage, 2001; Robey and Boskey, 2003). Transforming growth factor-1 β (TGF-1β) and IL-1β regulate the production of SPARC by chondrocytes at pre- and post-translational levels (Nakamura et al., 1996). In the absence of SPARC, mice develop progressive low turnover osteopenia. Trabecular bone volume is dramatically decreased, while cortical bone has compromised matrix quality (Delany et al., 2000; Boskey et al., 2003). SPARC-null mice showed decreased osteoblast and osteoclast, as well as reduced rate of bone formation. In addition, SPARC promotes osteoblastic commitment, differentiation, and survival, *in vitro* (Bradshaw et al., 2003; Delany et al., 2003; Robey and Boskey, 2003). Importantly, increased SPARC levels were reported in synovial fibroblasts from patients with RA or OA.

### Cardiovascular Diseases

In the heart, SPARC is mainly expressed by fibroblasts and endothelial cells, and to a lesser extent by cardiac myocardial cells (Chen et al., 2004; Harris et al., 2011). In addition, it has been well documented that SPARC is also overexpressed in atherosclerotic vascular lesions (Myasoedova et al., 2018). SPARC secretion occurs upon myocardial injury and at sites of cardiac remodeling (hypertrophy and fibrosis). In this context, Schellings et al. (2009) showed a SPARC-induced improvement of cardiac function following MI via regulation of post-synthetic procollagen processing in mice. Recently, Deckx et al. (2019) showed a novel inotropic function of SPARC in the heart. In particular, extracellular SPARC was found to directly elevate cardiomyocyte contraction and cardiac function both in healthy myocardium and during cardiac dysfunction induced by coxsackie virus, in *ex vivo* and *in vivo* studies (Deckx et al., 2019). The authors suggested a possible therapeutic application of SPARC when myocyte contractile function is diminished such as in myocarditis-related cardiac injury. SPARC also increases collagen cross-linking via its cysteine-rich repeats, and acts as a cofactor for TGF-β receptor signaling, leading to increased cardiac fibrosis. SPARC affinity for collagen was reported to be increased after its proteolytic cleavage by several MMPs, such as MMP-2, -3, -7, and -13 (Maurer et al., 1997). SPARC also regulates the activity of multiple growth factors including fibroblast growth factor 2 (Chen and Frangogiannis, 2010), PDGF (Motamed et al., 2002), and TGF-β (Francki et al., 2004; Schellings et al., 2009), which are crucially involved in fibrosis. However, despite SPARC-induced positive effect in the acute phase of MI, detrimental effects in pressure overload have been suggested to be induced by the increased SPARC-mediated collagen cross-linking (Bradshaw et al., 2009) and aging (Bradshaw et al., 2010), as it causes left ventricular diastolic dysfunction. In this context, monocytes and macrophages play a pivotal role in tissue fibrosis. SPARC is also expressed by inflammatory cells derived from bone marrow, suggesting a role for SPARC produced by infiltrating leukocytes in inflammatory response and fibrosis in the heart (Bradshaw, 2016). Therefore, SPARC appears to play a critical role in cardiac function and could be considered a potential therapeutic target.

## SFRP5

### Obesity and Metabolic Dysfunction

Secreted frizzled-related protein 5 is an antinflammatory adipokine secreted by WAT (Ouchi et al., 2010). It has been described as an endogenous blocker of WNT5A (wingless-type family member 5a signaling), an uncommon member of the WNT family proteins, involved in various cellular functions, such as inflammation, proliferation, differentiation, and migration (Kimura-Yoshida et al., 2005; Kim et al., 2010; Teo and Kahn, 2010; Neng et al., 2014). Although WNT5A was reported to be able to regulate cell proliferation and adipogenesis via MAPK, data on the relationships between WNT5A and SFRP5 in humans with obesity are limited and inconsistent (Hu W. et al., 2013; Lu et al., 2013; Miyoshi et al., 2014; Ji et al., 2017; Tang et al., 2018).

In 2012, Schulte and collaborators reported a significant increase of both WNT5A and SFRP5 concentrations in patients suffering from obesity compared with lean patients. In contrast, low plasma levels of SFRP5 have been detected in Chinese patients suffering from obesity (Hu Z. et al., 2013). Recently, Akoumianakis et al. (2019) found that circulating WNT5A concentrations were significantly higher in patients with obesity, paralleled by decreased plasma levels of SFRP5. Certainly, SFRP5 seems to play a critical role in obesity, as well as insulin resistance, and diabetes mellitus (Ouchi et al., 2010; Carstensen et al., 2013; Hu Z. et al., 2013; Cheng et al., 2015).

As regards to T2DM, it has been speculated that SFRP5 might prevent macrophage mediated inflammation of adipose tissue by antagonizing WNT5A protein with consequent improvement of insulin sensitivity. An important defensive function exerted by SFRP5 in the pathogenesis of T2DM has been described by Ouchi et al. (2010). In this context, mice deficient in SFRP5 (SFRP5−/−) fed a high-fat diet exhibited higher macrophage-mediated adipose tissue inflammation and insulin resistance respect to wild type mice due to uncontrolled activity of WNT5A. Conversely, in obese and diabetic mice, SFRP5 single dose administration enhanced metabolic function and reduced adipose tissue inflammation. The authors proposed that SFRP5 could neutralize the non-canonical activation of JNK implemented by WNT5a protein in macrophages and adipocytes through both paracrine and autocrine mechanisms (Ouchi et al., 2010).

Type 2 diabetes mellitus patients showed a decrease in plasma concentrations of SFRP5 which were unfavorably related to the evaluation of the homeostatic insulin resistance model, suggesting that SFRP5 could play a defensive action in the pathogenesis of T2DM (Hu Z. et al., 2013). By contrast, different studies have observed elevated SFRP5 plasma levels in T2DM patients (Lu et al., 2013; Canivell et al., 2015). Other studies are necessary to clarify the role of SFRP5 in obesity and metabolic syndrome.

### Rheumatic Diseases

Despite some data point to an involvement of WNT5A in the pathogenesis of RA, there is no clear evidence supporting a role of SFRP5 in rheumatic diseases. In particular, WNT5A enhanced gene expression of several cytokines that could be involved in RA progression (Blumenthal et al., 2006; Pereira et al., 2008; Halleskog et al., 2012; Rauner et al., 2012). In addition, higher WNT5A levels were detected in SF of patients with RA and OA (Sen et al., 2000) and its inhibition blocks the initiation of cultured synovial fibroblasts in patients with RA (Sen et al., 2001). As for SFRP5, it suppresses the inflammatory response in fibroblast-like synoviocytes of RA patients, inhibiting WNT signaling (Kwon et al., 2014).

### Cardiovascular Diseases

In spite of WNT5A has been particularly investigated in the progression of atherosclerosis (Pashirzad et al., 2017; Tong et al., 2019), studies on the relationship between WNT5A and SFRP5 in cardiovascular diseases are limited. Raised expression of WNT5A was reported in murine and human atherosclerotic lesions (Christman et al., 2008; Malgor et al., 2014). Recently, it has been suggested that SFRP5 could restore WNT5A-induced endothelial dysfunction (Cho et al., 2018).

An antinflammatory and antiapoptotic action for SFRP5 was also detected in a mouse model of ischemia/reperfusion injury (Nakamura et al., 2016), supporting a protective role on myocardial damage. Treatment of cardiomyocytes with angiotensin II is used to stimulate SFRP5, brain natriuretic peptide, and TNF-α, which induce cardiac hypertrophy (Jin et al., 2015).

Recently, it has been investigated the relationship between serum level of SFRP5 and severity of coronary artery disease. The results highlighted that serum SFRP5 was decreased in unstable coronary artery disease patients as compared to stable coronary artery disease patients and control subjects. Moreover, serum SFRP5 levels were inversely related to the presence and complexity of coronary artery disease (Safoura and Gholam, 2018).

More recently, Du et al. (2019) showed that high serum SFRP5 levels measured during the acute phase of ST segment elevation MI (STEMI) were significantly linked to early-stage myocardial recovery following primary percutaneous coronary intervention, suggesting that SFRP5 might represent a possible therapeutic target in acute STEMI.

## CTRPs

### Obesity and Metabolic Dysfunction

C1q/TNF-related proteins are a family of adipokines discovered in 2004 produced by different organs (Wong et al., 2004; Kopp et al., 2010b), and they have a structural similarity to adiponectin. CTRPs regulate energy homeostasis via adipoR1, adipoR2, and possibly other unknown receptors (Seldin et al., 2014). In particular, they modulate glucose and fat metabolism in peripheral tissues (Davis and Scherer, 2008; Wong et al., 2008; Peterson et al., 2010, 2012, 2014; Seldin et al., 2012, 2013; Wei et al., 2012, 2014; Petersen et al., 2017), food intake via a central mechanism (Byerly et al., 2013, 2014), inflammatory processes in adipose tissue (Enomoto et al., 2011), and adipocyte differentiation (Wei et al., 2013). On the other hand, much of the data regarding specific members are conflicting. Recently, preclinical and clinical studies have found that CTRP6 expression is upregulated in adipose tissue in both obesity and diabetic states (Lei et al., 2017). Stromal vascular cells and macrophages are among the main sources of CTRP6 (Kopp et al., 2010a). Increased expression of CTRP6 compromised glucose disposal of in peripheral tissues in mice. By contrast, deletion of the CTRP6 gene potentiated the effect of insulin and increased metabolism and energy expenditure in diet-induced obese mice (Lei et al., 2017). CTRP6 could regulate local inflammation and glucose metabolism by an action on macrophages and adipocytes. In particular, recombinant CTRP6 increased TNF-α expression and production in cultured macrophages, while decreased CTRP6 limited circulating inflammatory cytokines and pro-inflammatory macrophages in adipose tissue. In addition, CTRP6-overexpressing mice or CTRP6-treated adipocytes had decreased insulin-stimulated Akt phosphorylation and glucose uptake, while lack of CTRP6 improved Akt activation stimulated by insulin in adipose tissue. These effects suggest CTRP6 as a new metabolic/immune regulator linking obesity to inflammation and insulin resistance. Further complicating their role in obesity is that similar to adiponectin, CTRPs circulate in different isoforms that may contribute to their function in human diseases, but the current knowledge about the role of some CTRPs has not yet been fully elucidated.

### Rheumatic Diseases

In rheumatic disease, CTRP6 was found to regulate the activation of the complement system and its gene knock down worsens joint pathology in a mouse model of RA (Murayama et al., 2015). On the other hand, CTRP3 has also been recognized as a growth factor, promoting proliferation of chondrogenic precursors and chondrocytes (Maeda et al., 2006). In addition, it was reported that CTRP3 inhibits LPS-induced inflammatory cytokine production from human adipocytes, monocytes, and fibroblasts (Weigert et al., 2005; Kopp et al., 2010a; Hofmann et al., 2011), exerting an action in the development of CIA in mice (Murayama et al., 2014).

### Cardiovascular Diseases

CTRP1 is primarily secreted by stromal vascular cells, including adipose tissue macrophages, preadipocytes, and endothelial cells (Innamorati et al., 2002). CTRPs display significant activities in myocardial protection and vasodilation (Schäffler and Buechler, 2012). In fact, in heart, as well as in other tissue such as placenta, muscle, kidneys, prostate, and ovaries, was detected CTRP1 mRNA expression (Wang et al., 2016). The first works on cardiovascular disease described it as a potent antithrombotic factor, able to prevent collagen-induced platelet aggregation (Lasser et al., 2006). Recent evidences demonstrate that increased CTRP1 serum levels are protective against acute ischemic injury in coronary artery disease (Yuasa et al., 2016). Additional studies suggested the involvement of CTRP3 in the pathogenesis of MI (Yi et al., 2012; Choi et al., 2014; Lin et al., 2014; Wu et al., 2015). Immediately after a MI, a significant reduction of CTRP3 mRNA in adipose tissue and circulating levels of CTRP3 protein occurs in mice (Yi et al., 2012; Wu et al., 2015). In particular, CTRP3 causes mitochondrial biogenesis in cardiac tissue and vascular relaxation (Zhang et al., 2016). Exogenous CTRP3 pretreatment (adenovirus-delivered or recombinant CTRP3) showed increased preservation and improved cardiac function in animal models of MI (Wurm et al., 2007; Wu et al., 2015). In addition, CTRP3 has been hypothesized to be involved in progressive remodeling after MI (Wurm et al., 2007; Wu et al., 2015). On the other hand, CTRP9 was also described as a novel vasodilating adipokine that may produce vasoprotective actions via adiponectin receptor 1/AMPK/eNOS signaling pathway (Zheng et al., 2011). Recently, the relationships between serum CTRP9 levels and adhesion molecules in patients with T2DM and coronary artery disease have been investigated. It was found an important correlation between serum CTRP9 levels and adhesion molecules in coronary artery disease and T2DM patients, similarly to serum TNF-α levels in patients with coronary artery disease. Elevated circulating levels of CTRP9 in T2DM and coronary artery disease patients suggest a compensatory response aiming to restore a functional balance to insulin resistance, the inflammatory environment, and endothelial dysfunction in these patients (Moradi et al., 2018).

## FAM19A5

### Obesity and Metabolic Dysfunction

Family with sequence similarity to 19 member A5, known as TAFA5 is part of the TAFA family, secreted proteins expressed in the brain (Tom Tang et al., 2004).

Recent reports showed that FAM19A5 is released by subcutaneous, brown, epididymal, and perirenal adipose tissues (Wang et al., 2018). Interestingly, FAM19A5 levels in human adipocytes were significantly downregulated by TNF-α-induced inflammation (Tourniaire et al., 2013) suggesting that pro-inflammatory cytokines produced during obesity may cause FAM19A5 downregulation. However, differential expression of FAM19A5 in lean humans and individuals suffering from obesity has not been reported yet, and FAM19A5 could be regarded either a biomarker or a potential drug target of obesity-related cardiovascular disease in humans (Zarzour et al., 2018). A recent experimental study conducted on 223 individuals revealed that serum FAM19A5 concentrations were significantly elevated in patients with T2DM compared with subjects without T2DM. The plasma concentrations of FAM19A5 were positively correlated with waist circumference, waist–hip ratio, alanine aminotransferase, fasting plasma glucose, glycated hemoglobin, and mean brachial-ankle pulse wave velocity. Therefore, FAM19A5 could represent a biomarker of cardio-metabolic disease (Lee et al., 2019).

### Rheumatic Diseases

Despite several reports have shown that FAM19A5 is involved in various biological functions (Paulsen et al., 2008; Shahapal et al., 2019), studies on rheumatic disease are limited. FAM19A5 strongly stimulated mouse bone marrow-derived macrophages, resulting in chemotactic migration and inhibition of RANKL-induced osteoclastogenesis. The suppressive effect on osteoclastogenesis induced by FAM19A5 was completely reversed by a formyl peptide receptor antagonist (FPR) 2 WRW4 or by deficiency of FPR2, pointing to an important role of FPR2 in the regulation of osteoclastogenesis (Park M. Y. et al., 2017). These results suggest that FAM19A5 and its target receptor FPR2 could negatively regulate osteoclastogenesis. However, further works are necessary to elucidate its role in rheumatic diseases.

### Cardiovascular Diseases

It has been suggested that FAM19A5 might inhibit the proliferation and migration of smooth muscle cells (Wang et al., 2018). The authors also confirmed that FAM19A5 has been able to prevent post-injury neointima formation in rat carotid arteries and mouse femoral arteries *in vivo* (Wang et al., 2018). Given that FAM19A5 is expressed in human adipocytes and its mRNA expression and protein synthesis are downregulated by TNF-α (Tourniaire et al., 2013; Wang et al., 2018), it has been hypothesized that the production of FAM19A5 may be conditioned by cardio-metabolic disorders related to obesity in humans. Finally, Lee et al. (2019) suggested that FAM19A5 concentrations might reflect metabolic and cardiovascular status in humans. Further studies are needed to clarify its role in this disease.

## WISP1

### Obesity and Metabolic Dysfunction

Wingless-type inducible signaling pathway protein-1 (also known as CCN 4) is a component of the secreted protein associated with the ECM and a target gene of the WNT1 wingless pathway (Katoh and Katoh, 2007). WISP1 is released in various organs and tissues, such as brain, heart, pancreas, lung, intestine, kidney, ovaries, spleen, and displays anti-apoptotic effects through the PI3K and Akt pathways (Maiese et al., 2012). In addition, the signal transduction pathways of WISP1 are extensive, involving PI3K, PKB (Akt), mitogen MAPK, JNK, caspases, forkhead transcription factors, sirtuins, c-myc, glycogen synthase kinase-3β (GSK-3β), β-catenin, miRNAs, and the mechanistic target of rapamycin (mTOR) (Maiese, 2014). Murahovschi et al. (2015) showed that WISP1 is mainly found in visceral fat in patients with obesity and it is linked to markers of insulin resistance and inflammation in normal glucose tolerant patients. Murahovschi et al. (2015) showed that WISP1 is mainly found in visceral fat in patients with obesity and it is linked to markers of insulin resistance and inflammation in normal glucose tolerant patients. However, the specific function of WISP1 in the induction of insulin resistance is unclear. A study conducted on women with gestational diabetes and insulin resistance showed an increase in circulating WISP1 levels, which correlated with BMI and insulin resistance (Sahin Ersoy et al., 2016). More recently, it has been reported that WISP1 levels are increased in patients with obesity and are directly related to adiposity, regardless of glycemic status or insulin resistance; furthermore, they are highly correlated with increased plasma IL-8 (Barchetta et al., 2017). Ferrand et al. (2017) suggested that WISP1 could inhibit PPARγ activity. This evidence indicates that WISP1 acts as an unfavorable regulator of adipogenesis. On the other hand, a study published by Tacke et al. (2018) showed no differences in WISP1 levels among individuals with normal glucose tolerance, impaired glucose tolerance, and T2DM. However, a recent report suggested that WISP1 is strongly linked to the progression of insulin resistance, in particular higher circulating levels of WISP1 were found in patients suffering from obesity together with increased insulin resistance (Yaribeygi et al., 2019). Overall, WISP1 seems to play a critical role in many molecular pathways and cellular events; however, further studies are necessary to further deepen its involvement in obesity and metabolic disfunction.

### Rheumatic Diseases

Wingless-type inducible signaling pathway protein-1 plays a modulatory function on skeletal growth and bone repair (Yanagita et al., 2007). In particular, it modulates mesenchymal proliferation and osteoblastic differentiation and chondrogenic differentiation (French et al., 2004). Blom et al. (2009) have shown that the synthesis of WISP-1 was significantly intensified in the synovium and cartilage of mice with experimental OA, as well as in human OA cartilage and synovium. WISP1 regulates the expression of MMP and chondrocyte and macrophage aggrecanases with subsequent joint cartilage injury, in OA animal models (Blom et al., 2009). In addition, WISP-1 is involved in the regulation of osteogenesis through bone morphogenetic protein 2 (Ono et al., 2011). Considering its role in the control of cellular proliferation in the musculoskeletal system, WISP1 has been hypothesized as a potential therapeutic target in rheumatic diseases. On the other hand, Li H. H. et al. (2012) have linked WISP1 to the progression of inflammation, where recombinant WISP1 aggravates pro-inflammatory responses in LPS stimulated macrophages.

In this context, WISP1 is considered an important factor for the appearance of OA. In synovial fibroblasts of OA, WISP1 can stimulate the integrins αvβ5, PI3K, PKB (Akt), and NF-κB which drive upregulation of IL-6 production (Hou et al., 2013). In addition, WISP1 promoted chondrocyte hypertrophy through TGF-β signaling and activin-like kinase (ALK)1/Smad 1/5/8 pathway (van den Bosch et al., 2014). The detrimental effects of WISP1 in arthritic disease could be related to its role in promoting fibrosis (Berschneider et al., 2014; Jian et al., 2014).

In symptomatic humans suffering from OA with disease progression, higher WISP1 synovial expression was noted respect to non-progressing patients. In particular, WISP1 expression has been inversely related to the methylation levels of a positional CpG-dinucleotide (cg10191240), with damaged areas showing a significant hypomethylation for this CpG compared to preserved cartilage. Furthermore, it was noted that methylation levels were allele-dependent for a single intron nucleotide polymorphism near cg10191240. These results indicate that an increase in WISP1 is harmful to cartilage integrity in patients with OA (van den Bosch et al., 2019). The pathogenic role of WISP1 in OA has been also confirmed in Wisp1–/– mice (van den Bosch et al., 2017).

### Cardiovascular Diseases

In regards to cardiovascular diseases, various studies confirmed WISP1 positive effects on vascular and cardiovascular repair (Price et al., 2004; Reddy et al., 2011; Liu et al., 2013). It has been observed that WISP1 was upregulated after MI in animal models, and recombinant WISP1 was able to promote cell survival and collagen secretion in isolated myocytes and fibroblasts (Colston et al., 2007). WISP1 might regulate cardiac endothelial signaling and contribute to beneficial effects of post-MI treatments, as described by Wright et al. (2018). On the other hand, WISP1 seems able to rescue cardiomyocytes from doxorubicin toxicity (Lahoti et al., 2012), and prevent cardiomyocyte cell death through PI3K, Akt, and survivin (Venkatesan et al., 2010).

## Other Adipokines

Other adipokines, such as PRGN (also known as granulin/epithelin precursor), nesfatin-1 (nesfatin), visfatin/PBEF/NAMPT, apelin, RBP-4, and PAI-1 have been described as signal molecules involved in obesity and metabolic syndrome.

PROGRANULIN is a glycoprotein of approximately 75–80 kDa which is composed of seven granulin/epithelin repeats (granulins) (Wei et al., 2016). It can undergo enzymatic proteolysis into small homologous subunits. Both full-length protein and its constituent granulin peptides are biologically active with anti- and pro-inflammatory actions, respectively (Wei et al., 2016). PGRN is secreted by a broad spectrum of cells, including adipocytes, macrophages, and chondrocytes (Abella et al., 2017a). PGRN has been described as an adipokine with antinflammatory properties mediated by its competitive binding to TNF-α receptors (TNFR1 and TNFR2) (Abella et al., 2016, 2017b). Elevated PGRN expression in visceral adipose tissue and circulating PGRN concentrations are associated with visceral obesity, type 2 diabetes, and dyslipidemia (Youn et al., 2009; Qu et al., 2013; Shafaei et al., 2016). In particular, hyperprogranulinemia could be involved in the pathogenesis of obesity-associated insulin resistance, impairing insulin signaling and insulin-induced glucose uptake both *in vitro* and *in vivo*, whereas PGRN deficiency could protect from high fat diet-induced insulin resistance (Qu et al., 2013; Korolczuk and Bełtowski, 2017). In addition, different studies have demonstrated a link between hyperprogranulinemia, type 2 diabetes, and its renal, retinal, and microangiopathic complications (Xu et al., 2015; Albeltagy et al., 2019). Recent studies demonstrated that the loss of PGRN expression *in vivo* results in hypersusceptibility to collagen-induced arthritis, which can be reversed by the administration of recombinant PGRN (Tang et al., 2011). TNF-transgenic (TNF-Tg) mice with deletion of one or both alleles of PGRN exhibited significantly increased synovitis, pannus formation, destruction of the ankle joints, and loss of cartilage matrix (Tang et al., 2011). These data suggest that PGRN exerts its antinflammatory effects through inhibition of TNF/TNFR signaling *in vivo*. However, clinical studies have reported elevated PGRN levels in cartilage, SF, as well as serum from OA and RA patients (Cerezo et al., 2015; Abella et al., 2016), with elevated circulating PGRN levels significantly correlated to disease activity in RA patients (Yamamoto et al., 2014). Interestingly, the balance between PGRN and TNF seems to play a key role in RA progression, as the ratio PGRN/TNF is closely correlated with RA stages (Yamamoto et al., 2014). PGRN could be considered as a relatively new player in the pathogenesis of atherosclerosis. In this context, data suggest that PGRN might exert a role in the cross-talk of immunity and lipid turnover (Okura et al., 2010; de Jager and Pasterkamp, 2013). PGRN is also expressed by endothelial and T cells (Kawase et al., 2013; Abella et al., 2017a). In particular, PGRN improves tissue repair by active recruitment of leukocytes and increasing capillary perfusion after acute injury (Jian et al., 2012). It was found that PGRN mildly inhibits MCP-1-mediated monocyte migration, while it augments TNFα-mediated human aortic smooth muscle cell migration (Kojima et al., 2009). Additionally, PGRN inhibits TNFα-induced IL-8 release. It has been recently established that PGRN might have prognostic value in subclinical atherosclerosis (carotid artery intima media thickening), independent of other cardiovascular risk factors (Yoo et al., 2013).

NESFATIN-1 (nesfatin) is an N-terminal 82-amino-acid peptide nucleobindin-2-derived adipokine, involved in satiety induction and energy homeostasis. Nesfatin was first described as an anorexigenic molecule secreted by the hypothalamus (Oh et al., 2006), subcutaneous adipose tissue, gastric mucosa, pancreatic cells, and testes (Ayada et al., 2015). To date, its mechanism of action is not clarified. Central and peripheral nesfatin-1 injections inhibit food intake, in rodents (Cowley and Grove, 2006; Stengel and Taché, 2013), and a lack of nesfatin-1 in the brain leads to an increase of appetite and body fat and weight (Shimizu et al., 2009). Nesfatin-1-induced inhibition of feeding could be mediated through the inhibition of orexigenic neurons (García-Galiano et al., 2010; Stengel and Taché, 2010). In addition, nefastin-1 was found to regulate the activity of gastric distention-sensitive neurons and gastric motility via the melanocortin pathway in the central nucleus of amygdala (Wang et al., 2014). Various investigators suggested a possible role of nesfatin-1 in metabolic syndrome and T2DM (Gonzalez et al., 2011). Nesfatin-1 plays a role in obesity, hypertension, and diabetes through either the CNS or the peripheral sympathetic nervous system. The effects of nesfatin-1 on sympathetic nervous system activity could be a target for new anti-obesity drugs in leptin resistant people suffering from obesity (Masuo, 2014). In relation to rheumatic diseases, a study conducted by Kvlividze et al. (2019) demonstrated a positive association between blood serum nesfatin-1 levels and CRP in RA patients. Further studies showed a relationship between the concentration of nesfatin-1 and the severity of clinical manifestations of this disease, pointing to a pro-inflammatory effect of nesfatin-1 in the pathogenesis of RA. In addition, nesfatin-1 levels correlated with MMP-2 (Robinson et al., 2018). Nesfatin-1 has been detected in human and murine chondrocytes, where it was found to induce pro-inflammatory mediators such as COX-2, IL-8, IL-6, and CCL3 in chondrocytes from OA patients (Scotece et al., 2014a). Higher nesfatin-1 levels have also been detected in serum from OA patients compared to controls (Jiang et al., 2013). Moreover, serum and SF nesfatin-1 concentrations have been associated with radiographic severity of OA (Zhang et al., 2015), where its levels also correlated with CRP and IL-18 in serum and SF, respectively. In contrast to rheumatic desease, nesfatin-1 was described as an antinflammatory adipokine (Tang et al., 2012; Scotece et al., 2014a) which decrease cardiovascular risk (Dai et al., 2013; Ding et al., 2015). A few available studies show that this peptide affects energy metabolism of murine and human cardiomyocytes, by eliciting insulin-like effects. In rat heart, nesfatin-1 directly depresses contractility and relaxation via cGMP, PKG, and ERK1/2, and limits ischemia/reperfusion damage, displaying a post-conditioning protection. Nesfatin-1 actions are proposed to involve an unknown G-protein coupled receptor (Ramesh et al., 2017). However, in the rat heart, functional studies suggest an interaction with natriuretic peptide receptor-type A. These data open up novel perspectives to clarify not only the biological significance of nesfatin-1, but also its therapeutic potential in metabolic dependent cardiovascular diseases (Imbrogno et al., 2015). In addition, it has been observed that central and peripheral nesfatin-1 administration induces blood pressure elevation, probably mediated by α- and β-adrenergic receptors, respectively (Shimizu et al., 2009; Osaki and Shimizu, 2013). The authors speculated that nesfatin-1 might have a direct action on vascular smooth muscle, without involving the hypothalamic area (Shimizu et al., 2009; Osaki and Shimizu, 2013). However, recent reports suggest that nesfatin-1 may cause an increase in blood pressure in normotensive and hypotensive rats by increasing catecholamine, as well as vasopressin and renin levels (Aydin, 2013a,b). Finally, a recent study aimed to determine whether central cholinergic system may have a functional role in nesfatin-1-induced cardiovascular effect, in normotensive rats (Aydin et al., 2018).

### Visfatin/PBEF/NAMPT

A meta-analysis identified a significant increase of visfatin in patients diagnosed with overweight/obesity, T2DM, metabolic syndrome, and cardiovascular diseases (Chang et al., 2011). Additionally, it induces insulin resistance in many tissues and causes pancreatic beta cells dysfunction at later stages (Kumari and Yadav, 2018). These studies suggest that visfatin/PBEF/NAMPT could represent an important target to develop new drugs for metabolic and inflammatory diseases. In rheumatic diseases, visfatin/PBEF/NAMPT has been described as primarily a pro-inflammatory peptide (Meier et al., 2012; Neumann et al., 2016). In this context, visfatin/PBEF/NAMPT was found to be strongly related to various risk factors of both OA and OP, including obesity and diabetes mellitus. Interestingly, visfatin/PBEF/NAMPT has also been linked to several OA and OP pathologic features (Franco-Trepat et al., 2019). Visfatin/PBEF/NAMPT is a key factor for the progression of atherothrombotic disease and plaque destabilization by activating MMPs, promoting oxidation of lipoproteins by phospholipases, stimulating the release of acute phase proteins and pro-inflammatory cytokines (Anfossi et al., 2010). Moreover, visfatin/PBEF/NAMPT induces proliferation of vascular smooth muscle cells and fibroblasts, and plays a role in myocardial fibrosis and cardiac remodeling, in capillary tube neoformation mediated by the VEGF (Xiao et al., 2009).

APELIN has also been found in different tissues, such as brain, lung, kidney, bloodstream, and adipocytes (Lee et al., 2000; Kawamata et al., 2001; Boucher et al., 2005). Various studies confirmed that apelin plays a key role in energy metabolism and pathophysiology of obesity (Schinzari et al., 2017; Bertrand et al., 2018). Moreover, increased plasma apelin levels were found in individuals with obesity (Heinonen et al., 2005) as well as in those with type 2 diabetes (Li et al., 2006). A potential involvement of apelin in the pathogenesis of rheumatic diseases has been suggested, as confirmed by findings showing lower levels of this peptide in early state RA patients compared to healthy controls (Di Franco et al., 2012). More recently, apelin concentrations correlated positively with MMP-2 and inversely MMP-9, in RA patients (Gunter et al., 2017). Apelin was found to stimulate proliferation of chondrocytes and increase MMP-1, MMP-3, MMP-9, and IL-1β expression, *in vitro*. In agreement, increased expression of MMP-3, MMP-9, IL-1β, ADAMTS-4, and ADAMTS-5, as well as decreased level of collagen II were found following intra-articular injections of apelin, in rats. Proteoglycan in articular cartilage was also depleted following apelin treatment (Hu et al., 2010). A role for apelin in bone metabolism was also hypothesized, as confirmed by previous findings showing that apelin stimulated proliferation of osteoblasts through the APJ/PI3k/Akt pathway and exerted protective effects against apoptosis (Tang et al., 2007). On the other hand, increased apelin levels were found in both serum and SF of OA patients compared to healthy individuals (Hu et al., 2011). Both apelin and its receptor are expressed in vascular smooth muscle, endothelial, and myocardial cells (Maguire et al., 2009) and play key roles in regulation of cardiovascular functions (Yu et al., 2014). Also, plasma apelin concentrations are markedly reduced in patients with severe chronic heart failure (Chong et al., 2006). Apelin was also hypothesized to be critically involved in myocardial response to infarction and ischemia. In summary, clinical and experimental studies support a role for apelin system in cardiovascular diseases, making it an attractive therapeutic target.

### Retinol Binding Protein 4 (RBP4)

A relevant number of studies suggested an important link between RBP4 and adiposity (Aeberli et al., 2007; Haider et al., 2007; Jia et al., 2007; Klöting et al., 2007), insulin resistance (Gavi et al., 2007; Klöting et al., 2007; Perseghin et al., 2007; Stefan et al., 2007; Möhlig et al., 2008), type 2 diabetes (Cho et al., 2006; Takebayashi et al., 2007), and the metabolic syndrome (Qi et al., 2007). The effects of RBP4 could be mediated by retinoic acid receptors (RARs), retinoic acid-X receptors (RXRs), or receptors such as Megalin/gp320. Moreover, cAMP-mediated pathways, PPARγ and PPARα were found to be deeply involved in the regulation of RBP4 gene expression in BAT (Rosell et al., 2012). However, its metabolic effects on glucose metabolism in humans remain uncertain (von Eynatten and Humpert, 2008). In addition, Wei et al. (2019) demonstrated that elevated RBP4 serum levels were associated with increased risk of insulin resistance in patients with early and untreated AR. In patients with obesity, RBP4 could play a pivotal role in atherosclerotic process; however, the link between RBP4 and lipid profile needs further extensive research. To date, it is difficult to determine if RBP4 could constitute a novel biomarker useful in CVD (Rychter et al., 2020). At the time of this review, there is insufficient data regarding the contribution to CVD and RA.

### Plasminogen Activator Inhibitor-1 (PAI-1)

One other adipokine involved in obesity is PAI-1, a member of the serine protease inhibitor family. Various reports showed the complicated interaction between PAI-1, obesity, and metabolic disorders (Giltay et al., 1998). In particular, different studies demonstrated increased PAI-1 levels in subjects with obesity, metabolic syndrome, and T2DM (Vague et al., 1986; Landin et al., 1990; Juhan-Vague et al., 2003; Bilgili et al., 2008). Various reports described elevated PAI-1 levels in patients with systemic lupus erythematosus (SLE) and RA. In addition, individuals with SLE and RA showed a marked susceptibility in response to polymorphisms of PAI-1 (Angles-Cano et al., 1979; Glas-Greenwalt et al., 1984; Violi et al., 1990). However, more recently Bae and Lee (2020) demonstrated significantly increased circulating PAI-1 levels in patients with SLE but not in those with RA. On other hand, no association between PAI-1 4G/5G polymorphism and susceptibility to SLE and RA have been reported (Bae and Lee, 2020). In view of these results, it is clear that further studies are warranted to elucidate the direct contribution of PAI-1 in the pathogenesis of SLE and RA. Conversely, the connection between PAI-1 and cardiovascular events is well known. In particular, elevated plasma PAI-1 concentrations were found in both blood and coronary plaques of metabolic syndrome patients (Zorio et al., 2008). Similarly, elevated plasma PAI-1 levels have been associated with impaired fibrinolytic activity in stroke and CVD (Cesari et al., 2010; Jung et al., 2018).

## Conclusion

Experimental and clinical evidence links adipokine actions with obesity, metabolic dysfunction, rheumatic, and cardiovascular diseases. Considering the pleiotropic actions of these molecules and their metabolic, immunomodulatory, and miscellaneous effects both locally and systemically, the study of their function in the pathogenesis of these diseases is very complicated. Discordant results have been observed for many adipokines, with both pro- and antinflammatory effects reported for the same molecule, evidencing that their biological actions could be strictly dependent on the inflammatory context and disease status. Further research is needed to define the contribution of the adipokine network in these disorders, with the aim of identifying the adipokines with potential diagnostic and/or prognostic significance in managing obesity-associated diseases, such as metabolic dysfunction, rheumatic, and cardiovascular diseases.

## Author Contributions

LR, GO, LB, and SL contributed to the design and wrote the manuscript in consultation with CF and AC. All authors critically reviewed the manuscript.

## Conflict of Interest

The authors declare that the research was conducted in the absence of any commercial or financial relationships that could be construed as a potential conflict of interest.
